# A case report of sepsis associated coagulopathy after percutaneous nephrostomy

**DOI:** 10.1186/s12894-024-01476-x

**Published:** 2024-05-28

**Authors:** Juan Duan, Tao Ye, Yueyue Yang, Yiping Zhou, Shengyu Yang, Yueli Wang

**Affiliations:** Department of urology, 920thhospital of Joint Logistics Support Force, Kunming, PLA China

**Keywords:** sepsis, Hemorrhage, Coagulopathy, Ureteral calculus, Nephrostomy

## Abstract

**Background:**

Hemorrhage is a common complication of nephrostomy and percutaneous nephrolithotripsy, and it is caused by surgical factors. Here we report a rare case of hemorrhage caused by sepsis-related coagulation dysfunction.

**Case presentation:**

A 72-years-old male patient with bilateral ureteral calculi accompanied by hydronephrosis and renal insufficiency developed sepsis and hemorrhage on the third day after bilateral nephrostomy. After vascular injury was excluded by DSA, the hemorrhage was considered to be sepsis-associated coagulopathy(SAC/SIC), finally the patient recovered well after active symptomatic treatment.

**Conclusions:**

In patients with sepsis and hemorrhage, SAC/SIC cannot be excluded even if coagulation function is slightly abnormal after surgical factors are excluded. For urologists who may encounter similar cases in their general urology practice, it is important to be aware of these unusual causes of hemorrhage.

## Background

Sepsis-associated coagulopathy or sepsis induced coagulopathy (SAC/SIC) is one of the most common systemic dysfunctions in sepsis [1, 2]. Clinically evident coagulation dysfunction occurs in approximately 50–70% of septic patients, while 35% of these patients will develop into disseminated intravascular coagulation (DIC), the most severe state of SIC [3, 4]. The symptoms of SAC/SIC are diverse, ranging from mild thrombocytopenia to DIC [1, 5]. The diagnosis of SAC/SIC and DIC is primarily based on laboratory indicators, such as platelet count, PT, FDPs, fibrinogen and D-dimers [6–8]. Overt DIC is characterized by the elevated levels of FDPs or d-dimers, decreased platelet count, prolonged PT, and decreased fibrinogen levels [9]. Once diagnosed with DIC, clinical treatment is often unsuccessful, so it is important to identify SAC/SIC at early stage. The clinical manifestations and laboratory indicators of SAC/SIC are varied, ranging from mild coagulation activation of coagulation factor markers, to small decreases in platelet count and prolongation of subclinical total coagulation time, to sudden DIC characterized by both widespread microvascular thrombosis and massive bleeding at different sites [10]. The International Society on Thrombosis and Haemostasis (ISTH) DIC subcommittee suggested simple diagnostic criteria for sepsis induced coagulopathy (SIC) composed of only three parameters: plate count, PT or INR, and SOFA score [11]. Nevertheless, there are still some early and atypical SAC/SIC that cannot be diagnosed in a timely manner. In this paper, we report the diagnosis and treatment of a case of early postoperative bleeding caused by SAC/SIC after double kidney percutaneous nephrostomy, in order to enhance the vigilance of urologists on SAC/SIC, and to identify and treat some atypical SAC/SIC early.

## Case presentation

**Fig. 1:**
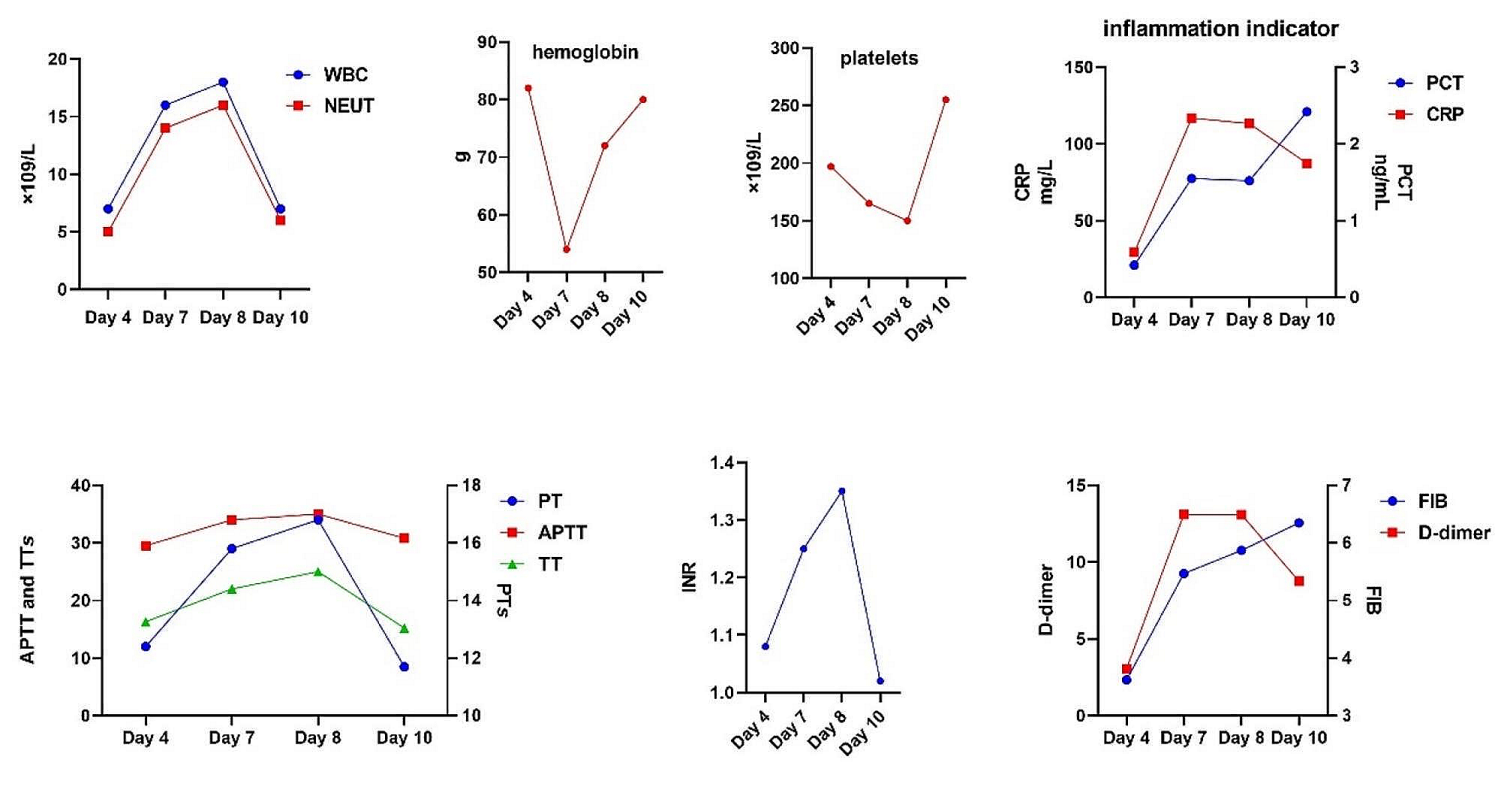
Changes in laboratory indicators during the disease process

**Fig. 2:**
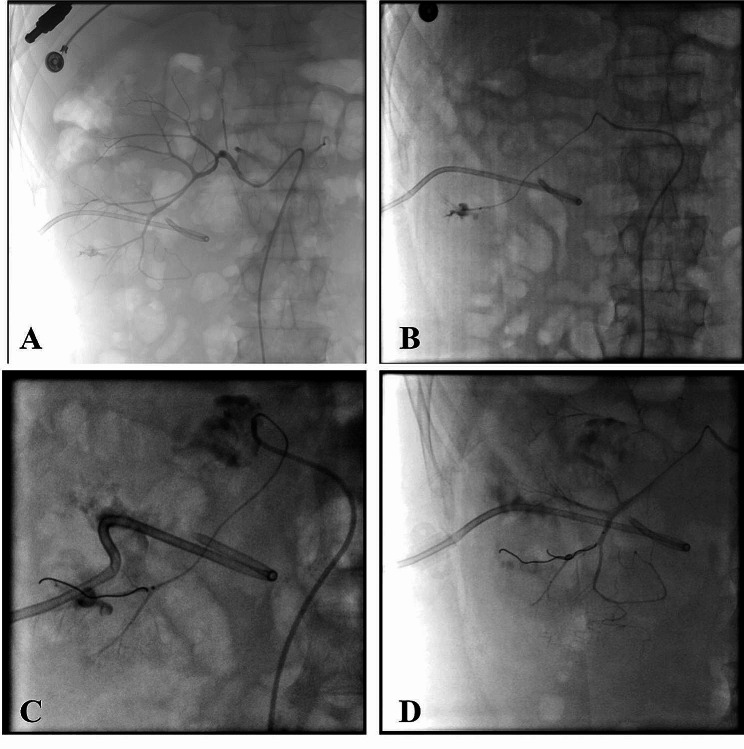
Detection and embolization of pseudoaneurysm through DSA **A** and **B** show the discovery of a pseudoaneurysm through DSA, **C** shows embolization of the pseudoaneurysm, **D**- shows there are no leakage of contrast agent after embolization

A 72-years-old male, was found to have elevated creatinine levels for more than 1 year and was admitted to the nephrology department after vomiting for 15 days. According to laboratory tests, creatinine was 1088µmol/L, urea was 36.77mmol/L, potassium was 5.31mmol/L and urine routine indicates white blood cells +++. The diagnosis of chronic renal insufficiency and urinary tract infection was made at day 1. The CT examination revealed calculi at the ends of both ureters and severe hydronephrosis of both ureters and kidneys, urine culture was positive for Enterococcus faecalis. Obstructive renal failure was the diagnosis. Hemodialysis was performed immediately, along with support treatment such as anti-infection (Cefoperazone and sulbactam sodium), blood transfusion and albumin supplementation. Local anesthesia was used for bilateral percutaneous nephrostomy at day 5. The preoperative blood routine tests showed: WBC 7.15 × 10^9^/L, NEUT 5.18 × 10^9^/L, NEUT% 72.4%, hemoglobin 85 g/L, and platelet 197 × 10^9^/L (Fig. 1). The surgical process went smoothly, the patents postoperative condition was stable, and the urine and drainage fluid were clear. However, the patient began to develop fever on the day 7, with a maximum body temperature of 39.2 degrees, rapid respiration of 25 times per minute, and blood pressure of 80/60mmHg. The urine and the nephrostomy fluid were both dark red, and about approximately 200 ml of dark red hematuria was discharged from the right nephrostomy. Laboratory examination showed 54 g/L hemoglobin, WBC 16.76 × 10^9^/L, NEUT14.55 × 10^9^/L, neutrophil 86.8%, platelet 165 × 10^9^/L, PT15.8s, INR1.25, ATPP34s, TT22s, FIB5.47 g/L, D dimer 13.1 mg/L, PCT1.52ng/ml (Fig. 1). The diagnosis implies sepsis accompanied by hemorrhage, and surgical factors cannot be exclude the cause of the hemorrhage. Unlike sepsis caused by ureteroscopy, which is mainly due to prolonged high-pressure perfusion, the patient in this case has renal insufficiency, combined with postoperative indwelling catheter and urine diversion. Anti-infection (Meropenem), fluid replacement, pressure boosting, oxygen inhalation, and red blood cell transfusion were all treatments given. During the same time (day 7), percutaneous selective renal angiography was performed, and it was found that a pseudoaneurysm in the right kidney was formed, which was accompanied by arterial embolism (Fig. 2). Even after embolization, the hemorrhage has not stopped after, and follow-up blood and coagulation tests show infection and coagulation disorders (Fig. 1). The cause of hemorrhage was considered SAC/SIC. In addition to continuing red blood cell transfusion, treatment also includes the meropenem for anti-infection, as well as platelet and cryoprecipitate transfusion and vitamin K. On the day 10, laboratory tests showed WBC 7.96 × 109/L, NEUT6.54 × 109/L, NEUT% 82.2%, PT11.7, INR1.02, TT15.2, APTT30.8, FIB6.35, D dimer 8.75 (Fig. 1). After the onset of fever, three consecutive urine cultures were positive for Enterococcus faecalis and Escherichia coli, thus increasing the anti-infection effect of linezolid and strengthening nutritional support treatment, the patient’s urine and drainage gradually clear up on the day 10. Half a month later, the patient’s condition stabilized and recovered well.

## Discussion and conclusions

Hemorrhage is the most common complication after nephrostomy, and its anatomical reasons include Pseudoaneurysm, arteriovenous fistula, and arterial laceration et al. All of the above factors can be clearly diagnosed and treated in the early stage through angiography [12–14]. SAC/SIC is difficult to identify and treat at early stage due to its atypical and diverse clinical manifestations [15]. In sepsis, there are several coagulation abnormalities including prolonged PT and APTT, high FDP and D-dimer, and exacerbation as the disease progresses. Thrombocytopenia is a more obvious manifestation of sepsis, and dynamic observation is more meaningful. The diagnosis of SAC/SIC is currentil lacking a relatively unified standard. Iba et al. proposed in 2017 to diagnose SAC/SIC through INR, PLT, and sequential organ failure assessment (SOFA) scoring systems [11]. This standard requires a total score of ≥ 4 points to diagnose SAC/SIC. In addition, Patrick et al. evaluated the value of the SAC/SIC score in predicting the risk of hospitalization death in a retrospective study [16]. The SAC/SIC score included two indicators, PLT and PT-INR, and sepsis patients were divided into four levels based on INR and PLT: mild, moderate, severe SAC/SIC, and no SAC/SIC. Mild SAC/SIC is defined as INR ≥ 1.2 and < 1.4, plus platelet count ≤ 150,000/µL but > 100,000/µL; Moderate SAC/SIC is defined as INR ≥ 1.4 but < 1.6 or platelet count ≤ 100,000/µL but > 80,000/µL; Severe SAC/SIC is defined as INR ≥ 1.6 and platelet count ≤ 80,000/µL [16]. All this shows early diagnosis of SAC/SIC is difficult.

We report a case of postoperative hemorrhage after percutaneous nephrostomy, in which there were only slight abnormal changes in laboratory coagulation related indicators. We consider the presence of SAC/SIC, after ruling out surgical causes of bleeding, which mainly manifest as coagulation disorders. We blood transfusions, along with cryoprecipitates, platelets and vitamin K treatment were administered proactively, and the patient’s recovery was successful. The research shows that SAC/SIC is closely linked to the risk of death in sepsis patients [17–19]. The diagnosis of SAC/SIC solely based on coagulation related indicators still has significant shortcomings and is prone to missed diagnosis [20]. Although the patient reported here has coagulation dysfunction, it cannot be diagnosed according to the above diagnostic criteria. After excluding surgical factors, we diagnosed SIC and actively intervened. The patient ultimately achieved a good prognosis.

SAC/SIC is a serious complication of sepsis. Early diagnosis of SAC/SIC and timely intervention are very important. The diagnostic criteria for SAC/SIC are not perfect. We considered that after excluding other factors, those who can not be diagnosed according to the current diagnostic criteria, but there are hemorrhage or extensive coagulation disorders, SAC/SIC cannot be completely excluded, and early intervention should be carried out.

## Data Availability

All data generated or analysed during this study are included in this published article.

## Abbreviations

SAC/SIC: Sepsis-associated coagulopathy or sepsis induced coagulopathy
DSA: Digital subtraction angiography
DIC: Disseminated intravascular coagulation
PT: Prothrombin time
INR: International standardized ratio
APTT: Active partial thromboplastic time
FIB: Fibrinogen
TT: Thrombin time
FDP: Fibrinogen degradation products
